# Supplementary material for: Algal photosystem I dimer and high-resolution model of PSI-plastocyanin complex
Source: Nat Plants. 2022 Oct 13;8(10):1191–201. doi: 10.1038/s41477-022-01253-4 (PMC9579051; doi:10.1038/s41477-022-01253-4)
Supplement: Supplementary file 1 — Reporting Summary [file 41477_2022_1253_MOESM1_ESM.pdf]

nature portfolio

Corresponding author(s): ALEXEY AMUNTS

Last updated by author(s): Aug 26, 2022

Reporting Summary

Nature Portfolio wishes to improve the reproducibility of the work that we publish. This form provides structure for consistency and transparency in reporting. For further information on Nature Portfolio policies, see our Editorial Policies and the Editorial Policy Checklist.

Statistics

For all statistical analyses, confirm that the following items are present in the figure legend, table legend, main text, or Methods section.

n/a	Confirmed	
☐	☒	The exact sample size (n) for each experimental group/condition, given as a discrete number and unit of measurement
☐	☒	A statement on whether measurements were taken from distinct samples or whether the same sample was measured repeatedly
☒	☐	The statistical test(s) used AND whether they are one- or two-sided<br>*Only common tests should be described solely by name; describe more complex techniques in the Methods section.*
☒	☐	A description of all covariates tested
☒	☐	A description of any assumptions or corrections, such as tests of normality and adjustment for multiple comparisons
☒	☐	A full description of the statistical parameters including central tendency (e.g. means) or other basic estimates (e.g. regression coefficient) AND variation (e.g. standard deviation) or associated estimates of uncertainty (e.g. confidence intervals)
☒	☐	For null hypothesis testing, the test statistic (e.g. F, t, r) with confidence intervals, effect sizes, degrees of freedom and P value noted<br>*Give P values as exact values whenever suitable.*
☒	☐	For Bayesian analysis, information on the choice of priors and Markov chain Monte Carlo settings
☒	☐	For hierarchical and complex designs, identification of the appropriate level for tests and full reporting of outcomes
☒	☐	Estimates of effect sizes (e.g. Cohen's d, Pearson's r), indicating how they were calculated

Our web collection on statistics for biologists contains articles on many of the points above.

Software and code

Policy information about availability of computer code

Data collection	The datasets were collected EPU 1.9 software on FEI Titan Krios (FEI/Thermofischer) transmission electron microscope operated at 300 keV with a slit width of 20 eV on a GIF quantum energy filter (Gatan). A K2 Summit detector (Gatan) was used at a pixel size of 0.84 Å (magnification of 105,000x) with an exposure rate of 15.27 electrons/pixel/second fractionated over 45 frames. A defocus range of -0.7 to -2.5 µm was used.
Data analysis	Movie frames were aligned and averaged by global and local motion corrections by MotionCor2. Contrast transfer function (CTF) parameters were estimated by CTFFIND4. Particles were picked and initially 2D classified by RELION 3.1. 2D classification and 3D heterogeneous refinement steps were performed in cryoSPARC v.3.1. The models were manually built with Coot 0.914 and stereochemical refinement was performed using phenix.real_space_refine in the PHENIX 1.17.1 suite. The final figures were generated using UCSF Chimera 1.14, UCSF Chimera X 0.91, Pymol 2.0.<br>Mass-spectrometry data was processed in MaxQuant 1.6.14, Perseus 1.6.15.0, Proteome Discoverer 2.4.<br>Spectra were normalized to the maximum of the emission peaks using the Jasco spectra analysis program Spectra Manager II.

For manuscripts utilizing custom algorithms or software that are central to the research but not yet described in published literature, software must be made available to editors and reviewers. We strongly encourage code deposition in a community repository (e.g. GitHub). See the Nature Portfolio guidelines for submitting code & software for further information.

Data

Policy information about availability of data

All manuscripts must include a data availability statement. This statement should provide the following information, where applicable:

- Accession codes, unique identifiers, or web links for publicly available datasets
- A description of any restrictions on data availability
- For clinical datasets or third party data, please ensure that the statement adheres to our policy

The atomic coordinates were deposited in the RCSB Protein Data Bank (PDB). The local resolution filtered maps, half maps, masks and FSC-curves have been deposited in the Electron Microscopy Data Bank (EMDB).
Mass spectrometry datasets: Project Name: Intensity-based absolute quantification (iBAQ) of components of photosystem I monomers and dimers. Project accession: PXD026990. Project DOI: 10.6019/PXD026990. Project Name: Identification of photosystem I components from Chlamydomonas reinhardtii grown under oxic and anoxic conditions. Project accession: PXD027067. Project name "Label-free quantification of photosystem I monomers and dimers from Chlamydomonas reinhardtii." Project accession: PXD028024. Project DOI: 10.6019/PXD028024.

Human research participants

Policy information about studies involving human research participants and Sex and Gender in Research.

Reporting on sex and gender	*Use the terms sex (biological attribute) and gender (shaped by social and cultural circumstances) carefully in order to avoid confusing both terms. Indicate if findings apply to only one sex or gender; describe whether sex and gender were considered in study design whether sex and/or gender was determined based on self-reporting or assigned and methods used. Provide in the source data disaggregated sex and gender data where this information has been collected, and consent has been obtained for sharing of individual-level data; provide overall numbers in this Reporting Summary. Please state if this information has not been collected. Report sex- and gender-based analyses where performed, justify reasons for lack of sex- and gender-based analysis.*
Population characteristics	*Describe the covariate-relevant population characteristics of the human research participants (e.g. age, genotypic information, past and current diagnosis and treatment categories). If you filled out the behavioural & social sciences study design questions and have nothing to add here, write "See above."*
Recruitment	*Describe how participants were recruited. Outline any potential self-selection bias or other biases that may be present and how these are likely to impact results.*
Ethics oversight	*Identify the organization(s) that approved the study protocol.*

Note that full information on the approval of the study protocol must also be provided in the manuscript.

Field-specific reporting

Please select the one below that is the best fit for your research. If you are not sure, read the appropriate sections before making your selection.

☒ Life sciences ☐ Behavioural & social sciences ☐ Ecological, evolutionary & environmental sciences

For a reference copy of the document with all sections, see nature.com/documents/nr-reporting-summary-flat.pdf

Life sciences study design

All studies must disclose on these points even when the disclosure is negative.

Sample size	A total of 17,439 movies were recorded and analyzed. No statistical analyses has been performed. The number of cryo-EM particles in the single dataset collected was the number of particles available. No predetermined sample size was used for other experiments.
Data exclusions	Particles that were not photosystem I were excluded in the analysis, since they cannot contribute to reconstruction.
Replication	Similar cryo-EM structures were successfully obtained from preliminary datasets.
Randomization	Particle images were randomly assigned into half-sets to obtain gold-standard resolution estimates as described in the text.
Blinding	N/A to cryo-EM study; raw micrographs or particle images are not categorical data. Particles are randomly assigned into half-sets for image processing; hence no blinding is applicable.

Reporting for specific materials, systems and methods

We require information from authors about some types of materials, experimental systems and methods used in many studies. Here, indicate whether each material, system or method listed is relevant to your study. If you are not sure if a list item applies to your research, read the appropriate section before selecting a response.

Materials & experimental systems

n/a	Involved in the study
☐	☒ Antibodies
☒	☐ Eukaryotic cell lines
☒	☐ Palaeontology and archaeology
☒	☐ Animals and other organisms
☒	☐ Clinical data
☒	☐ Dual use research of concern

Methods

n/a	Involved in the study
☒	☐ ChIP-seq
☒	☐ Flow cytometry
☒	☐ MRI-based neuroimaging

Antibodies

Antibodies used	primary rabbit anti-PsaF, -Pc, -PsaA, -Lhca5, -Lhca2, -Lhca9, -Lhca3, -PsaD, -PsaG, -Lhcb/a, PsbA and LhcSR3 as referenced in the text<br>secondary horseradish peroxidase (HRP) conjugated goat anti-rabbit IgG (H + L) (Bio-Rad)
Validation	validation provided by manufacturer
