# Supplementary material for: Algal photosystem I dimer and high-resolution model of PSI-plastocyanin complex
Source: Nat Plants. 2022 Oct 13;8(10):1191–201. doi: 10.1038/s41477-022-01253-4 (PMC9579051; doi:10.1038/s41477-022-01253-4)
Supplement: Supplementary file 4 — Chemical moieties involved in coordination of chlorophyll a/b. [file 41477_2022_1253_MOESM4_ESM.pdf]

Supplementary Table 1. Chemical moieties involved in coordination of chlorophyll *a*/*b*.

	Chlorophyll *a*/*b*										
	His	Met	1x H_2O	2x H_2O	Gln	back-bone	Lipid	Asp	Glu	Asn	Total
**All subunits**	84	2	13/1	22/19	13	11/7	10	5/3	28	11	199/30
**PsaA**	32	1	3	4	3	1	1				45
**PsaB**	31	1	5		1		1	1			40
**PsaF**				2				1			3
**PsaG**	1							1			2
**PsaJ**									1		1
**PsaK**	1			1		1		1			4
**PsaL**	1		1								2
**Lhca1_a**	2			3/2		1/1	1		3	1	11/3
**Lhca1_b**	2		1/1	2/1		1/1	1		3	1	11/3
**Lhca3**	2		1	2/1	2	2			3	1	13/1
**Lhca4**	1			1/3	1	0/1	1	1/1	3	2	10/5
**Lhca5**	3			2/3	1	2	1	0/1	3	1	13/4
**Lhca6**	3		1	1/3	1	0/2	1	0/1	3	1	11/6
**Lhca7**	2		1	2/2	1	1/1	1		3	1	12/3
**Lhca8**	2			2/2	1	1/1	1		3	1	11/3
**Lhca9**	1			0/2	2	1	1		3	2	10/2
