# Supplementary material for: Algal photosystem I dimer and high-resolution model of PSI-plastocyanin complex
Source: Nat Plants. 2022 Oct 13;8(10):1191–201. doi: 10.1038/s41477-022-01253-4 (PMC9579051; doi:10.1038/s41477-022-01253-4)

Source Data related to Extended Data Figure 1

Coomassie stain presented in ED Fig. 1c
(Marker bands are at approximately 180, 130, 100, 70, 55, 40, 35, 25, 15 and 10 kDa.)

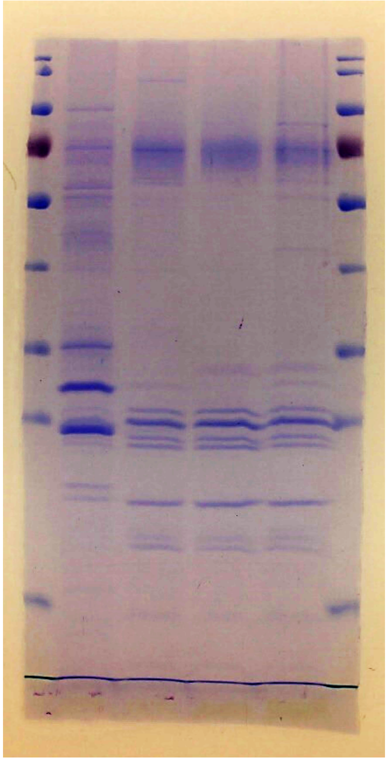

Western Blot against PsaF presented in ED Fig. 1d
(Marker bands are at approximately 180, 130, 100, 70, 55, 40, 35, 25, 15 and 10 kDa.)

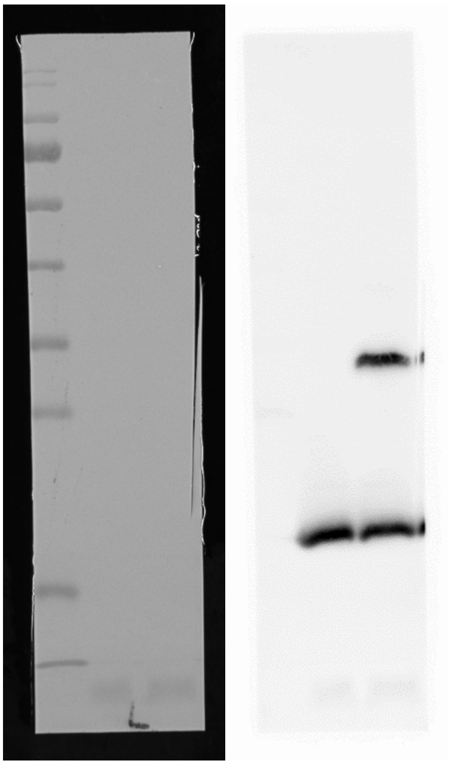

Western Blot against PsaA presented in ED Fig. 1d
(Marker bands are at approximately 180, 130, 100, 70 and 55 kDa.)

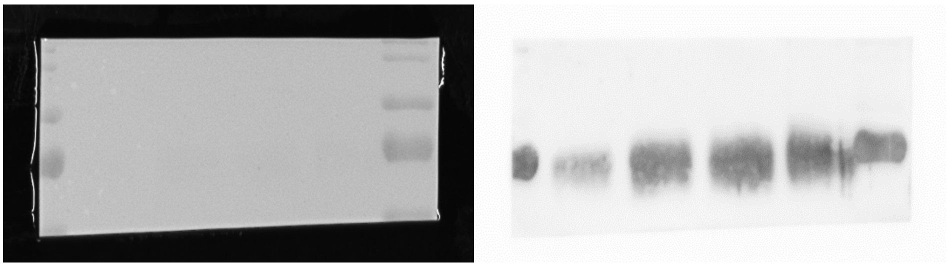

Western Blot against Lhca5 presented in ED Fig. 1d
(Marker bands are at approximately 35 and 25 kDa.)

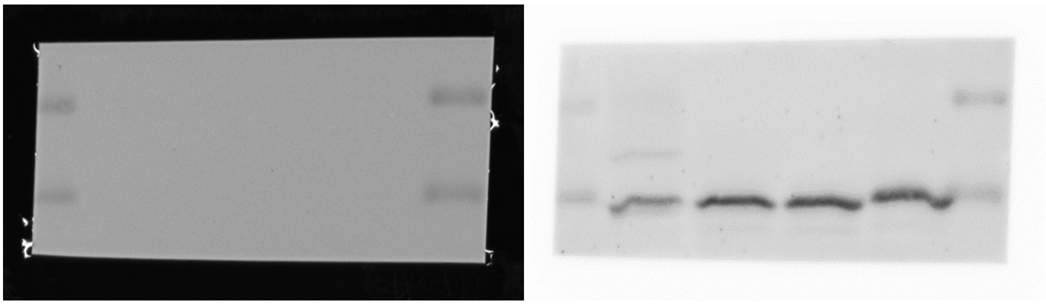

Western Blot against Lhca2 presented in ED Fig. 1d
(Marker bands are at approximately 35 and 25 kDa)

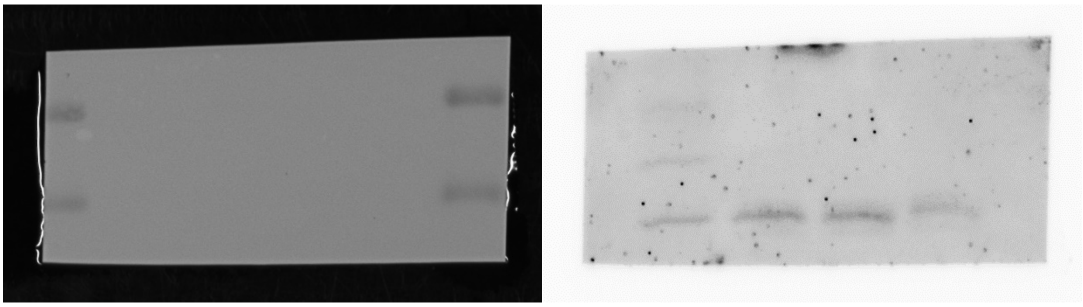

Western Blot against Lhca9 presented in ED Fig. 1d
(Marker band is at approximately 15 kDa.)

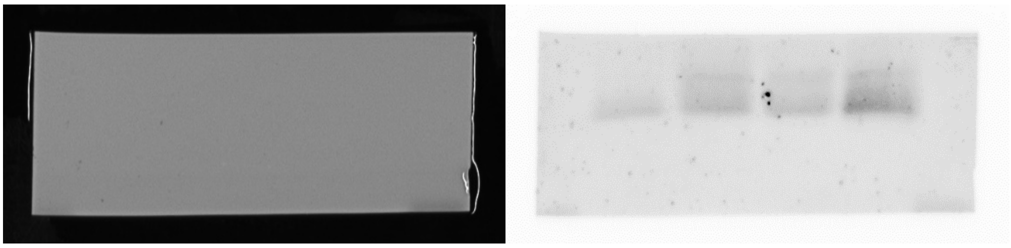

Western Blot against PsaD presented in ED Fig. 1d
(Marker band is at approximately 15 kDa.)

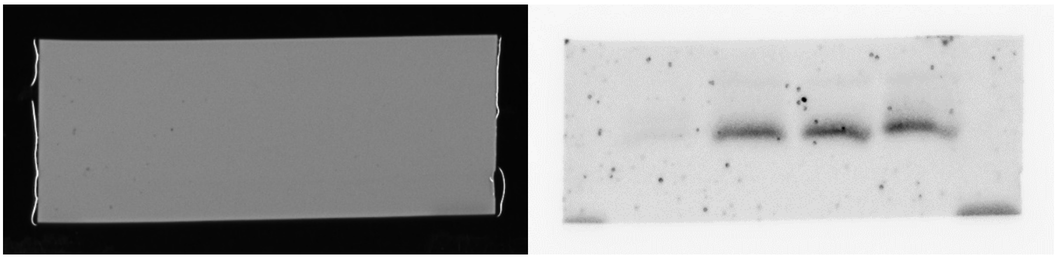

Western Blot against PsaG presented in ED Fig. 1d
(Marker bands are at approximately 15 and 10 kDa.)

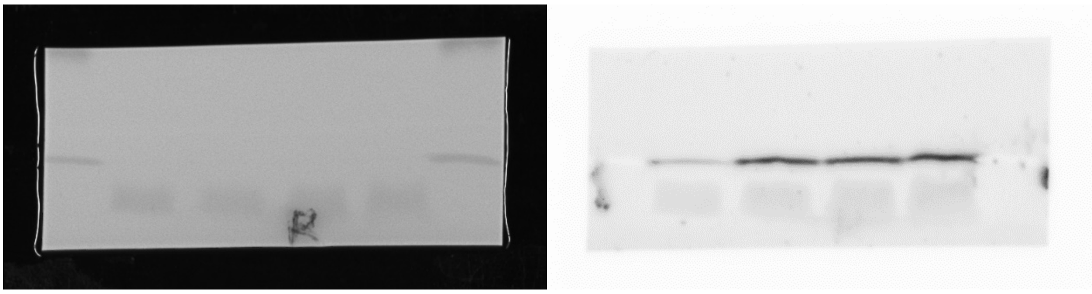

Silver stain presented in ED Fig. 1i

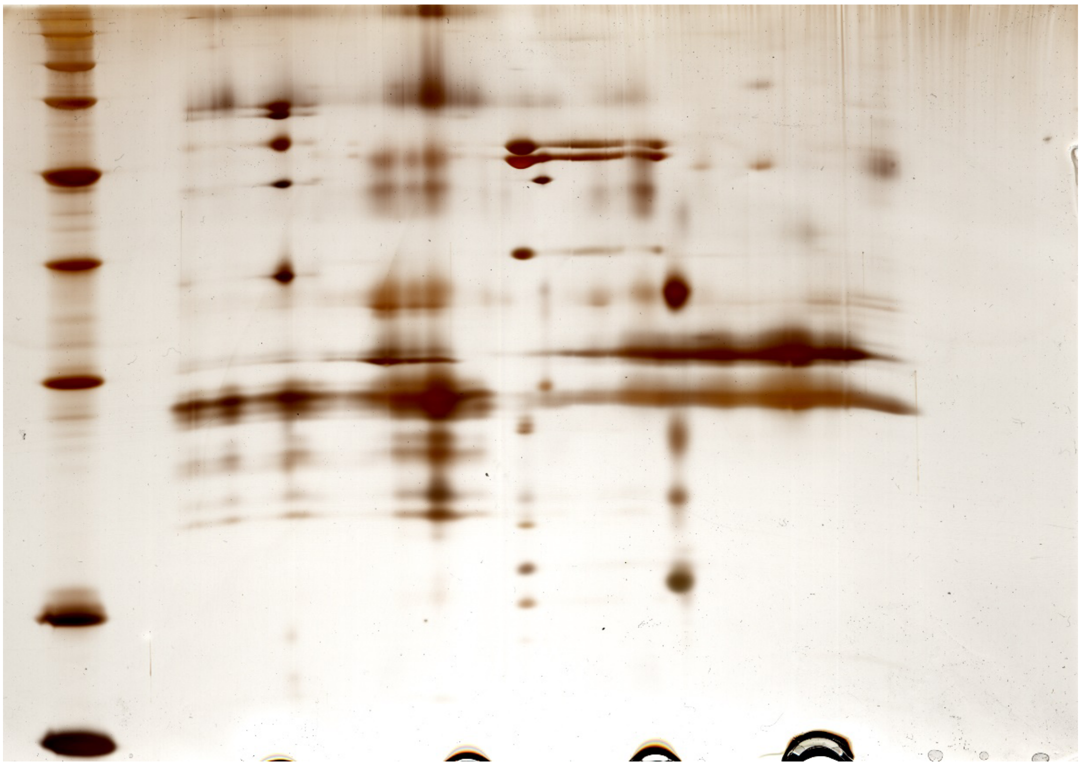

Western Blot against Lhca3 and PsaD presented in ED Fig. 1i
(Additional signals are related to PsbA, which was probed on the same membrane.
The membrane was also incubated with anti-LhcSR3 (no signal).)

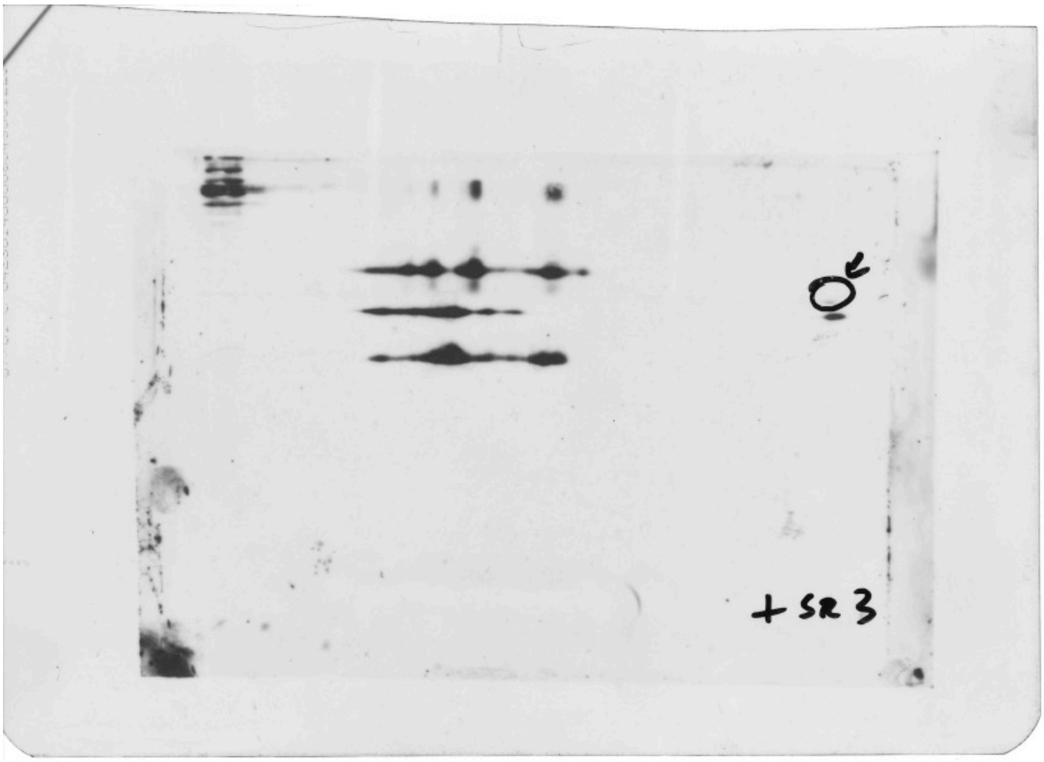

Western Blot against Lhcb/a presented in ED Fig. 1i

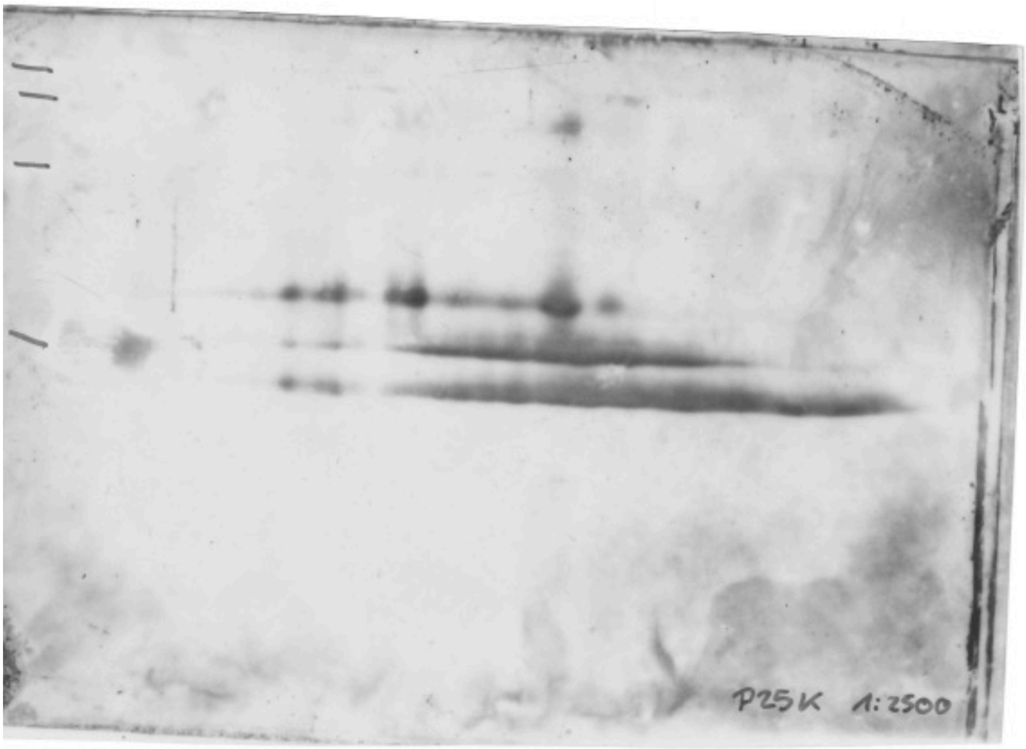

Silver stain presented in ED Fig. 1j

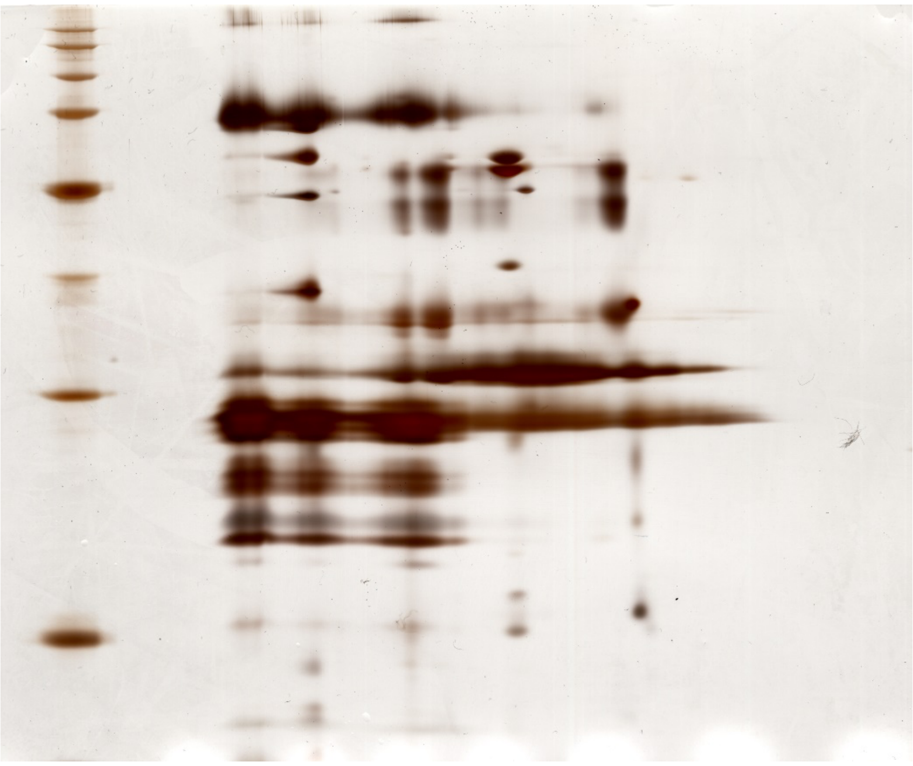

Western Blot against Lhca3 and PsaD presented in ED Fig. 1j
(The membrane was also incubated with anti-LhcSR3 (no signal).)

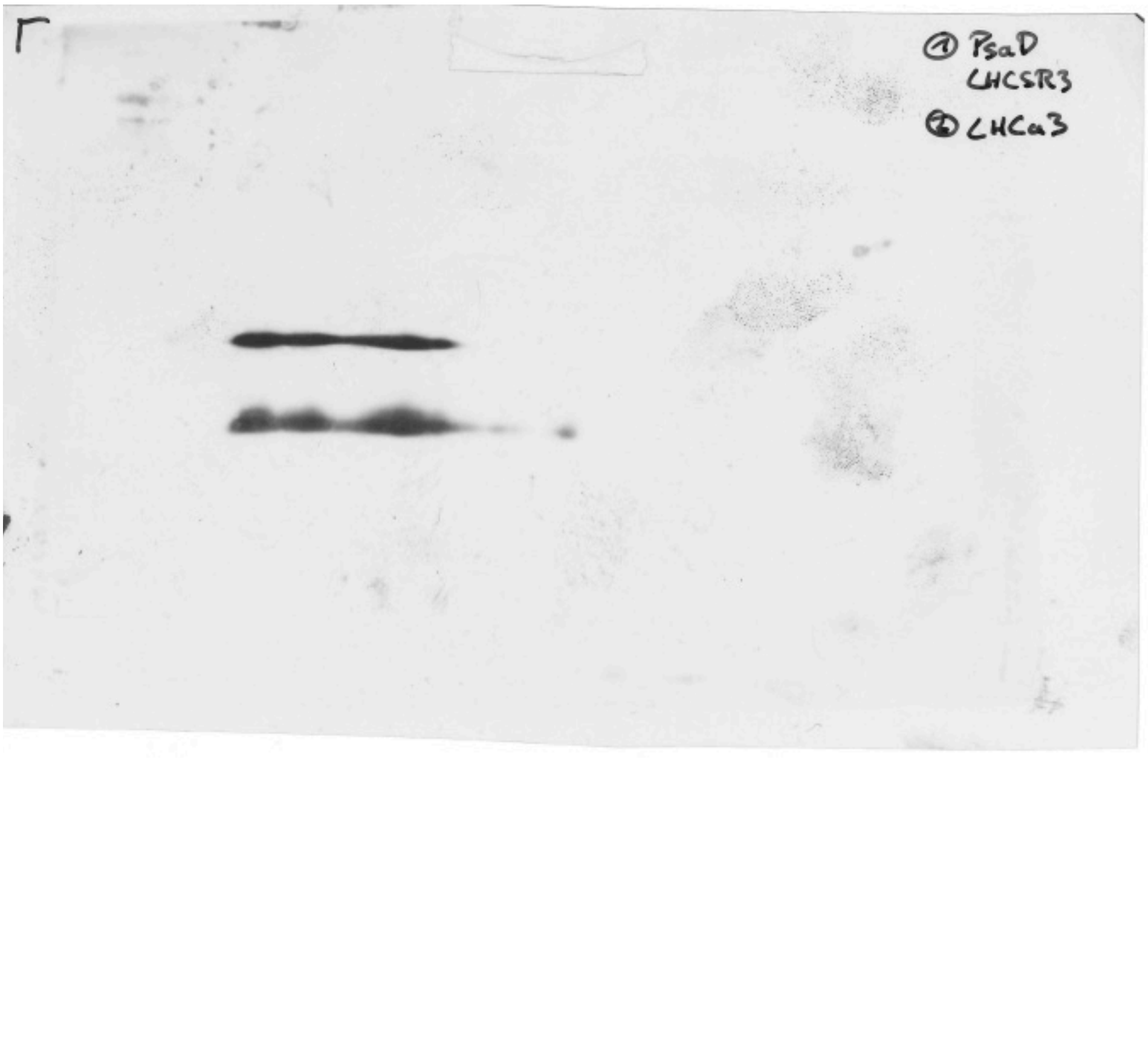

Western Blot against Lhcb/a presented in ED Fig. 1j
(Additional signals are related to PsaD, which was probed on the same membrane.
The membrane was also incubated with anti-LhcSR3 (no signal).)

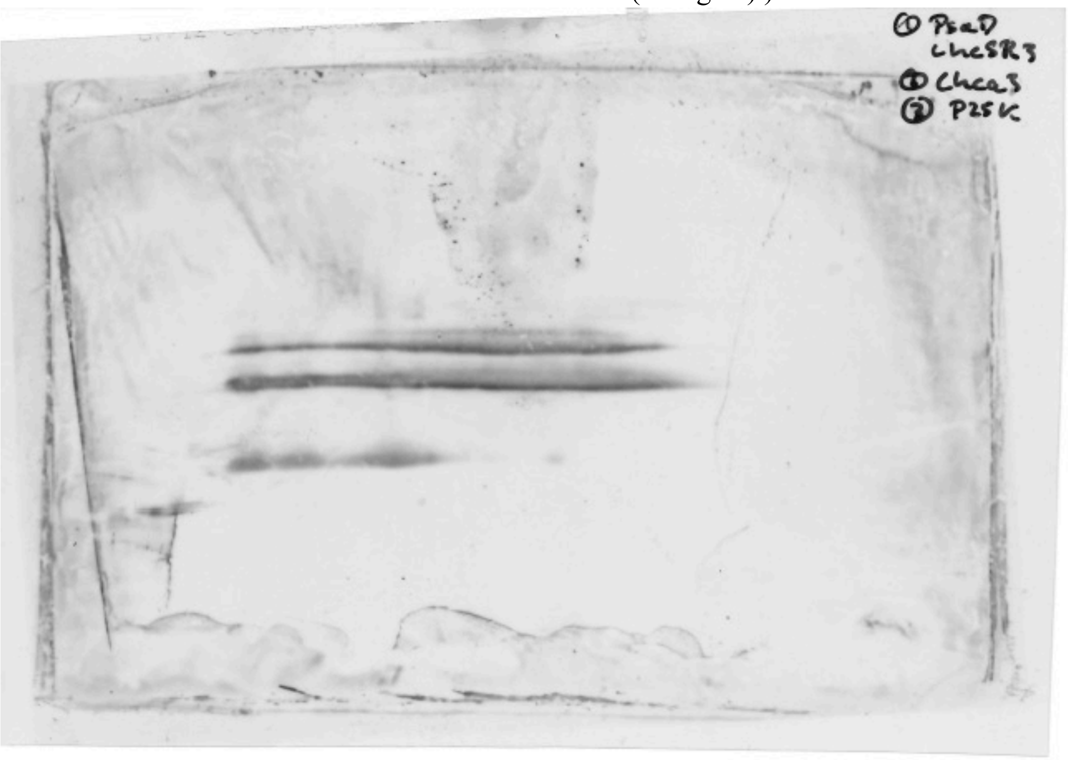

Supplement: Supplementary file 5 — Unmodified Coomassie stains, western blots and silver stains for Fig. 1. [file 41477_2022_1253_MOESM5_ESM.pdf]
